# Supplementary material for: Enhancing the Antimicrobial Effect of Ozone with Mentha piperita Essential Oil
Source: Molecules. 2023 Feb 21;28(5):2032. doi: 10.3390/molecules28052032 (PMC10004731; doi:10.3390/molecules28052032)
Supplement: Supplementary file 1 [file molecules-28-02032-s001.zip › molecules-2059921-supplementary.pdf]

Table S1. Optical density (OD) values at 540 nm for experimental variants.

	C. albicans		*E. coli*		*P. aeruginosa*		*S. aureus*		*S. mutans*	
	**Ozone**	**Ozone + MpEO**	**Ozone**	**Ozone + MpEO**	**Ozone**	**Ozone + MpEO**	**Ozone**	**Ozone + MpEO**	**Ozone**	**Ozone + MpEO**
5''	1.537 ± 0.004^{a}	0.193 ± 0.003^{b}	1.537 ± 0.004^{a}	0.256 ± 0.002^{b}	1.537 ± 0.004^{a}	0.272 ± 0.003^{b}	1.537 ± 0.004^{a}	0.268 ± 0.002^{b}	1.537 ± 0.004^{a}	0.257 ± 0.002^{b}
10''	1.469 ± 0.006^{a}	0.185 ± 0.003 ^{b}	1.469 ± 0.006^{a}	0.241 ± 0.003^{b}	1.469 ± 0.006^{a}	0.266 ± 0.002^{b}	1.469 ± 0.006^{a}	0.197 ± 0.058^{b}	1 .469 ± 0.006^{a}	0.242 ± 0.003^{b}
15''	1.381 ± 0.003^{a}	0.176 ± 0.002^{b}	1.381 ± 0.003^{a}	0.220 ± 0.003^{b}	1.381 ± 0.003^{a}	0.257 ± 0.002^{b}	1.381 ± 0.003^{a}	0.194 ± 0.004^{b}	1.381 ± 0.003^{a}	0.229 ± 0.003^{b}
20''	1.364 ± 0.002^{a}	0.171 ± 0.002^{b}	1.364 ± 0.002^{a}	0.220 ± 0.003^{b}	1.364 ± 0.002^{a}	0.241 ± 0.003^{b}	1.364 ± 0.002^{a}	0.185 ± 0.003^{b}	1.364 ± 0.002^{a}	0.223 ± 0.002^{b}
25''	1.350 ± 0.004^{a}	0.170 ± 0.001^{b}	1.350 ± 0.004^{a}	0.213 ± 0.002^{b}	1.350 ± 0.004^{a}	0.213 ± 0.002^{b}	1.350 ± 0.004^{a}	0.174 ± 0.002^{b}	1.350 ± 0.004^{a}	0.202 ± 0.002^{b}
30''	1.345 ± 0.002^{a}	0.169 ± 0.001^{b}	1.345 ± 0.002^{a}	0.190 ± 0.002^{b}	1.345 ± 0.002^{a}	0.190 ± 0.002^{b}	1.345 ± 0.002^{a}	0.147 ± 0.003^{b}	1.345 ± 0.002^{a}	0.184 ± 0.003^{b}
35''	1.337 ± 0.004^{a}	0.168 ± 0.002^{b}	1.337 ± 0.004^{a}	0.187 ± 0.002^{b}	1.337 ± 0.004^{a}	0.187 ± 0.002^{b}	1.337 ± 0.004^{a}	0.142 ± 0.002^{b}	1.337 ± 0.004^{a}	0.164 ± 0.002^{b}
40''	1.324 ± 0.002^{a}	0.167 ± 0.002^{b}	1.324 ± 0.002^{a}	0.175 ± 0.002^{b}	1.324 ± 0.002^{a}	0.175 ± 0.002^{b}	1.324 ± 0.002^{a}	0.139 ± 0.001^{b}	1.324 ± 0.002^{a}	0.147 ± 0.001^{b}
45''	1.324 ± 0.003^{a}	0.166 ± 0.001^{b}	1.324 ± 0.003^{a}	0.167 ± 0.003^{b}	1.324 ± 0.003^{a}	0.167 ± 0.003^{b}	1.324 ± 0.003^{a}	0.137 ± 0.002^{b}	1.324 ± 0.003^{a}	0.143 ± 0.002^{b}
50''	1.219 ± 0.003^{a}	0.164 ± 0.001^{b}	1.219 ± 0.003^{a}	0.160 ± 0.002^{b}	1.219 ± 0.003^{a}	0.160 ± 0.002^{b}	1.219 ± 0.003^{a}	0.131 ± 0.002^{b}	1.219 ± 0.003^{a}	0.140 ± 0.002^{b}
55''	1.118 ± 0.002^{a}	0.162 ± 0.002^{b}	1.118 ± 0.002^{a}	0.156 ± 0.003^{b}	1.118 ± 0.002^{a}	0.156 ± 0.003^{b}	1.118 ± 0.002^{a}	-	1.118 ± 0.002^{a}	0.220 ± 0.002^{b}
60''	1.113 ± 0.004^{a}	0.160 ± 0.002^{b}	1.113 ± 0.004^{a}	0.155 ± 0.002^{b}	1.113 ± 0.004^{a}	0.155 ± 0.002^{b}	1.113 ± 0.004^{a}	-	1.113 ± 0.004	-
120''	1.104 ± 0.002^{a}	0.137 ± 0.002^{b}	1.104 ± 0.002^{a}	0.130 ± 0.003^{b}	1.104 ± 0.002^{a}	0.130 ± 0.003^{b}	1.104 ± 0.002^{a}	-	1.104 ± 0.002	-
180''	1.075 ± 0.002	-	1.075 ± 0.002	-	1.075 ± 0.002	-	1.075 ± 0.002	-	1.075 ± 0.002	-
240''	1.003 ± 0.002	-	1.003 ± 0.002	-	1.003 ± 0.002	-	1.003 ± 0.002	-	1.003 ± 0.002	-
Control	1.322 ± 0.002^{a}	0.712 ± 0.003^{b}	1.322 ± 0.002^{a}	0.665 ± 0.002^{b}	1.322 ± 0.002^{a}	0.452 ± 0.005^{b}	1.322 ± 0.002^{a}	0.429 ± 0.002^{b}	1.322 ± 0.002^{a}	-

The results are presented as mean values ± standard deviations. $^{a–b}$The mean differences between the samples were compared using a t-test. Data for the same strain at the same time (in a row) with various superscripts are significantly different ($p < 0.05$).

Table. S2. BGR%/MGR% of ozone.

TIME (s)	*S. mutans*	*S. aureus*	*P. aeruginosa*	*E. coli*	*C. albicans*
5"	116.24	114.75	116.91	100.19	129.85
10"	111.09	113.51	116.24	99.84	128.72
15"	104.44	110.42	115.20	99.36	127.62
20"	103.20	109.18	114.90	98.90	125.61
25"	102.09	104.67	111.75	98.58	124.14
30"	101.74	103.38	111.26	98.39	123.80
35"	101.13	101.97	110.59	98.02	122.59
40"	100.18	101.30	110.13	97.48	117.38
45"	100.18	100.11	109.46	96.43	115.83
50"	92.23	95.27	108.36	96.70	112.72
55"	84.54	76.46	108.24	95.95	110.08
60"	84.22	65.20	107.33	88.60	106.30
120"	83.48	60.98	96.46	82.92	98.40
180"	81.29	50.01	49.02	67.18	92.74
240"	75.87	19.09	45.79	60.93	74.81
Control	99.99	100.00	100.03	99.97	100.04

Table S3. BGR%/MGR% of ozone enhanced with MpEO.

TIME (s)	*S. mutans*	*S. aureus*	*P. aeruginosa*	*E. coli*	*C. albicans*
5"	116.97	116.97	60.25	38.55	27.11
10"	110.15	110.15	58.78	36.29	26.03
15"	104.24	104.24	56.78		24.72
20"	101.36	101.36	53.39		24.06
25"	91.67	91.67	47.05		23.92
30"	83.64	83.64	42.11		23.74
35"	74.70	74.70	41.30		23.60
40"	66.97	66.97	38.79		23.41
45"	64.85	64.85	36.87		23.31
50"	63.48	63.48	35.47		23.03
55"			34.44		22.75
60"			34.29		22.52
120"			28.76		19.29
Control	100.00	100.00	99.93	99.95	99.95

Table S4. BGR%/MGR% of MpEO on ATCC strains.

	S. mutans	*S. aureus*	*P. aeruginosa*	*E. coli*	*C. albicans*
MpEO 2μL	63.41	66.45	51.67	37.91	32.46
MpEO 4μL	60.53	65.27	47.29	36.51	35.67
MpEO 8μL	58.40	65.16	39.88	32.29	42.30
MpEO 16μL	50.88	41.85	34.51	29.34	43.08
MpEO 32μL	49.87	32.20	32.40	25.94	48.93
Control	100.00	100.05	100	100	100.00

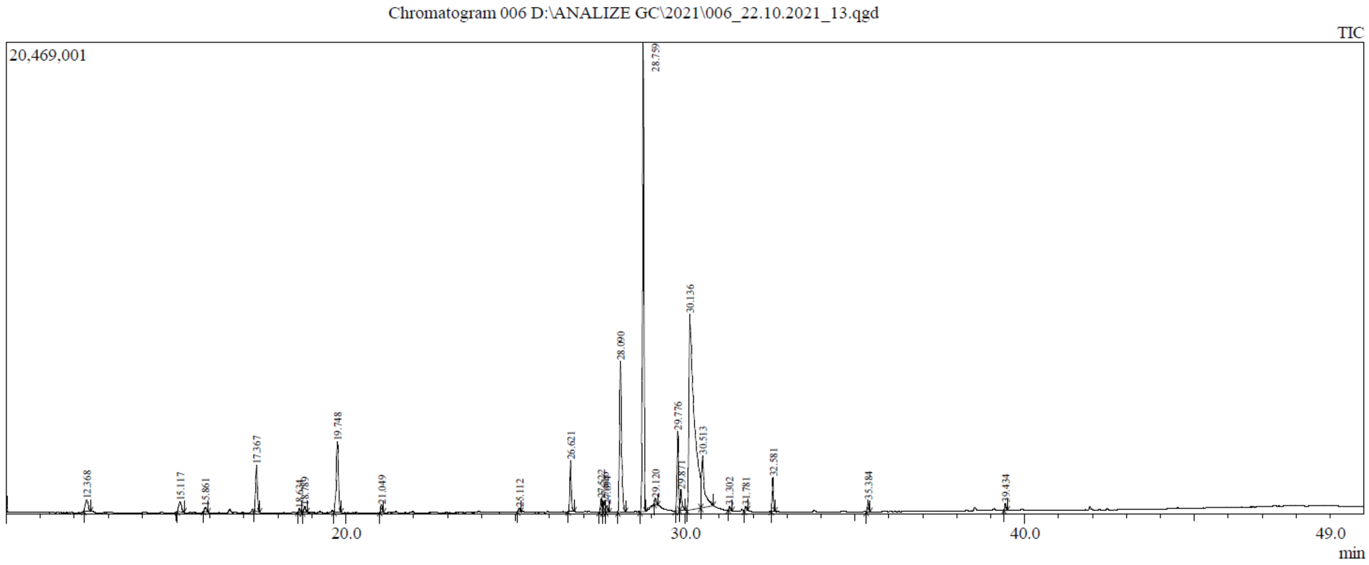

Figure S1. GC-MS Chromatogram.

Quantitative Result Table

ID#	Name	R.Time	m/z	Area	Height	Conc.	Conc.Un
1	.alpha.-Pinene	12.368	TIC	3849320	567932	1.476 %	
2	.beta.-Pinene	15.117	TIC	3074475	481953	1.179 %	
3	Sabinene	15.861	TIC	1325842	252820	0.508 %	
4	L-Limonene	17.367	TIC	8179439	2061810	3.137 %	
5	beta.-trans-Ocimene	18.634	TIC	607045	181033	0.233 %	
6	gamma.-Terpinene	18.789	TIC	1041191	282413	0.399 %	
7	Eucalyptol	19.748	TIC	14703694	3096877	5.639 %	
8	p-Cymol	21.049	TIC	1182739	357326	0.454 %	
9	Menthofuran	26.621	TIC	7719224	2276216	2.960 %	
10	Linalool	27.522	TIC	1960197	593382	0.752 %	
11	Linalool acetate	27.616	TIC	1455409	462189	0.558 %	
12	Cyclohexanol, 5-methyl-2-(1-methylethyl)-, ac	27.694	TIC	979036	315676	0.375 %	
13	Menthyl acetate racemic	28.090	TIC	26915480	6582569	10.322 %	
14	p-Menthan-3-one,	28.759	TIC	61652571	20364881	23.643 %	
15	Menthol, acetate, iso-	29.120	TIC	4907455	550156	1.882 %	
16	Isomenthone	29.776	TIC	10549899	3465782	4.046 %	
17	4-Terpineol	29.871	TIC	3104653	890589	1.191 %	
18	Menthol	30.136	TIC	85134942	8435941	32.648 %	
19	Germacrene D	30.513	TIC	14106301	2260228	5.410 %	
20	.gamma.-Elemene	31.302	TIC	636958	189422	0.244 %	
21	p-menth-1-en-8-ol	31.781	TIC	1127940	242201	0.433 %	
22	Pulegone	32.581	TIC	4266982	1520684	1.636 %	
23	Piperitone	35.384	TIC	1358661	492795	0.521 %	
24	Caryophyllene oxide	39.434	TIC	925360	318679	0.355 %	
